# Obesity and Sympathetic Overactivity in Young Individuals With Hypertension: Clinical Perspective of Indian Healthcare Providers

**DOI:** 10.7759/cureus.74115

**Published:** 2024-11-20

**Authors:** Uday Jadhav, Dharmesh Solanki, Srinivas Kumar, Prakash Hazra, Thomas Alexander, Amit Gupta, Shweta Ghatge, Santosh Revankar

**Affiliations:** 1 Cardiology, Mahatma Gandhi Mission (MGM) New Bombay Hospital, Navi Mumbai, IND; 2 Interventional Cardiology, Wockhardt Hospital, Rajkot, IND; 3 Cardiology, Apollo Health City, Hyderabad, IND; 4 Cardiology, AMRI Hospitals, Kolkata, IND; 5 Cardiology, Kovai Medical Center and Hospital (KMCH), Coimbatore, IND; 6 Medical Affairs, USV Private Limited, Mumbai, IND; 7 Scientific Services, USV Private Limited, Mumbai, IND

**Keywords:** beta-blockers, coronary artery disease, hypertension, metoprolol, obesity, preventive effect, sympathetic overactivity, telmisartan

## Abstract

Introduction: To understand the current clinical practices followed by healthcare professionals (HCPs) among populations with hypertension and obesity with sympathetic overactivity and develop strategies to improve the management of hypertension.

Methods: A standard questionnaire was formulated based on high sympathetic overactivity and/or obesity in young patients with hypertension to gather information on the perception and practices of HCPs toward the management of young patients with hypertension who have high sympathetic overactivity and/or obesity. HCPs throughout India were selected. The key insights and recommendations from the panel discussion were summarized in a report that helped to develop strategies to improve the management of young hypertension patients with high sympathetic overactivity/obesity.

Results: A total of 1170 HCPs participated in the survey and in panel discussion meetings. According to 53% of HCPs, patients with hypertension with increased sympathetic overactivity or stress are most commonly aged 41-60 years. These patients have a higher likelihood of developing stroke (60%), alcoholism (46%), and sleep apnea (41%). Moreover, these HCPs also opined that patients with hypertension and obesity are at greater risk of developing coronary artery disease and chronic kidney disease (69%) and often require multiple antihypertensive drugs (60%). For the management of hypertension in obese patients with sympathetic overactivity, a combination of telmisartan and cardio-selective beta-blockers is the preferred treatment (66%). Additionally, a combination of telmisartan and metoprolol is recommended to control sympathetic overactivity in obese patients with hypertension.

Conclusion: Sympathetic overactivity is becoming more common in young adults with hypertension, and the combination of telmisartan and cardio-selective beta-blockers is the best treatment option for these patients. This approach may help to effectively manage hypertension and reduce the risk of complications associated with sympathetic overactivity. The limitation of the study is its reliance on self-reported data from HCPs, which may introduce bias.

## Introduction

Hypertension is a serious medical condition associated with an increased risk of heart, brain, kidney, and other diseases. According to the data from the National Health and Nutrition Examination Survey (NHANES) from August 2021 to August 2023, the prevalence of hypertension among adults was 47.7%, with a rate of 23.4% specifically for those aged 18-39 years [1]. Over 128 billion adults aged 30-79 years suffer from hypertension globally, most of them (two-thirds) living in low- and middle-income countries [2]. According to reports, India has a high prevalence of hypertension ranging between 25% and 42% [3,4], where the reported incidence among young adults (20-39 years age group) was around 11% [5]. For individuals aged 18 to 39 years, hypertension is defined as having a systolic blood pressure (SBP) or diastolic blood pressure (DBP) of 130/80 mmHg or higher, or being on antihypertensive medication [6]. Experiencing hypertension during young adulthood can result in early end-organ damage, heighten the risk of premature and lifelong cardiovascular disease, and negatively impact both productivity and quality of life [7]. High blood pressure during young adulthood causes vascular damage, which leads to clinical events and mortality later in life [8].

The prevalence of obesity has reached epidemic proportions. In India, the prevalence of obesity varies from 11.8% to 31.3% [9]. One important mechanism of increased body weight with high blood pressure is sympathetic overactivation. A recent review article suggests that sympathetic overdrive may not immediately affect blood pressure, but increases in visceral fat are an important determinant of an increase in blood pressure [10].

Sympathetic overload is involved in the pathogenesis of hypertension by modifying heart rate, cardiac output, peripheral vascular resistance, and renal sodium retention [11]. The NHNES found that there is a link between hypertension and insulin resistance. As insulin resistance increases, the activity of the sympathetic nervous system and the retention of sodium in the body also increase. Additionally, obesity is also associated with insulin resistance and inflammation, both of which can contribute to hypertension [12]. Moreover, several epidemiological studies have found a correlation between insulin resistance and hypertension in early adulthood [13,14]. This indicates that the two conditions are closely related and that sympathetic overactivation is a common factor in adult patients with both insulin resistance and hypertension.

There is a gap in the evidence for the optimal treatment of hypertension in younger individuals. Many clinical trials focus on patients over 50 years old, leaving a lack of evidence for the most effective treatments for younger individuals with hypertension [15]. Several societies, including the European Society of Cardiology/European Society of Hypertension (ESC/ESH) and International Society for Hypertension (ISH), and Indian guidelines on hypertension-IV (2019) have recommended the use of angiotensin-converting enzyme inhibitors or angiotensin II receptor blockers (ARBs) as a first-line therapy for treating hypertension [16-18].

Beta-blockers have not been fully utilized in the treatment of hypertension. Beta-blockers may be crucial in managing hypertension in young adults due to sympathetic overactivity, linked to hypertension development in India [19].

A joint statement of the European Association for the Study of Obesity and the European Society of Hypertension reported that non-selective beta-blockers might increase body weight. However, cardio-selective beta-blockers that reduce cardiac output and renin activity, either alone or in combination, have been shown to effectively lower blood pressure, particularly in young, obese patients [20].

Many guidelines recommend using beta-blockers in specific situations such as angina, post-myocardial infarction, and heart failure with reduced ejection fraction [16-18]. A recent cross-sectional study from India reported that most clinicians prefer using a combination of ARBs and beta-blockers as the first-line treatment for managing hypertension in young adults. Beta-blockers are particularly favored as the drug of choice due to their effectiveness in addressing sympathetic overactivity commonly observed in this population [21].

Despite the widespread use of beta-blockers in the young Indian population with hypertension and high sympathetic overactivity, there is limited data on their effectiveness in this population. This expert viewpoint aims to gain insight into the perception and practices of healthcare professionals (HCPs) regarding the management of young patients with hypertension with high sympathetic overactivity and to evaluate the role of telmisartan and metoprolol in treating this population.

## Materials and methods

An expert panel of cardiologists collaborated to design a survey questionnaire aimed at assessing hypertension management in young adults (aged < 40 years) who worked on the following points: incidence and clinical conditions associated with sympathetic overactivity; challenges in the treatment of hypertension in obese; management of sympathetic overactivity and obesity.

A survey was conducted according to the globally accepted standards of good clinical practice (as defined in the International Conference on Harmonization E6 Guidelines for Good Clinical Practice, 1st May 1996) [22]. The questionnaire is listed in the Appendix. The survey was conducted online using Google Forms Google (Mountain View, CA) and was rolled out to 950 HCPs across India over a two-month period (May 2022 to July 2022). A total of 828 participants responded to the survey, and their responses were recorded.

During the virtual meetings, a diverse panel of experts (n = 342) from different geographical regions of India discussed and provided their opinions on all survey questions. After analyzing the survey questions and consulting with the expert panelists, clinical insights were extracted and compiled to create this expert viewpoint.

This study was conducted according to the Declaration of Helsinki principles [23] and followed the guidelines for Good Epidemiology Practice [24]. The ACEAS Independent Ethics Committee approved the study on 15 October 2022.

Statistical analysis

All responses to the survey questionnaires were analyzed and entered into a suitable spreadsheet. Qualitative variables were represented as frequency and percentage. A P-value < 0.05 was considered as statistically significant.

## Results

A total of 828 HCPs participated in an online survey, and 342 participated in a round table discussion. A panel of experts (n = 442, 53%) opined that the age group 41-60 years was identified as the most common age group with sympathetic overactivity/stress (Table 1). Patients with hypertension who have increased sympathetic overactivity often have a higher likelihood of developing stroke (n = 500, 60%), alcoholism (n = 381, 46%), and sleep apnea (n = 337, 41%). These were reported as the most common clinical conditions associated with increased sympathetic overactivity (Table 1).

**Table 1:** Common age group and clinical conditions with high blood pressure and sympathetic overactivity. Data are presented as n (%).

Parameter	No. of doctors (N = 828)
Age group	
21 to 40 years	358 (43.24)
41 to 60 years	442 (53.38)
>60 years	28 (3.38)
Clinical conditions	
Stroke	500 (60.39)
Sleep apnea	337 (40.70)
Thyroid problem	304 (36.71)
Alcoholism	381 (46.01)
Paroxysmal hypertension	348 (42.03)
Others (diabetes)	8 (0.97)

**Figure 1:**
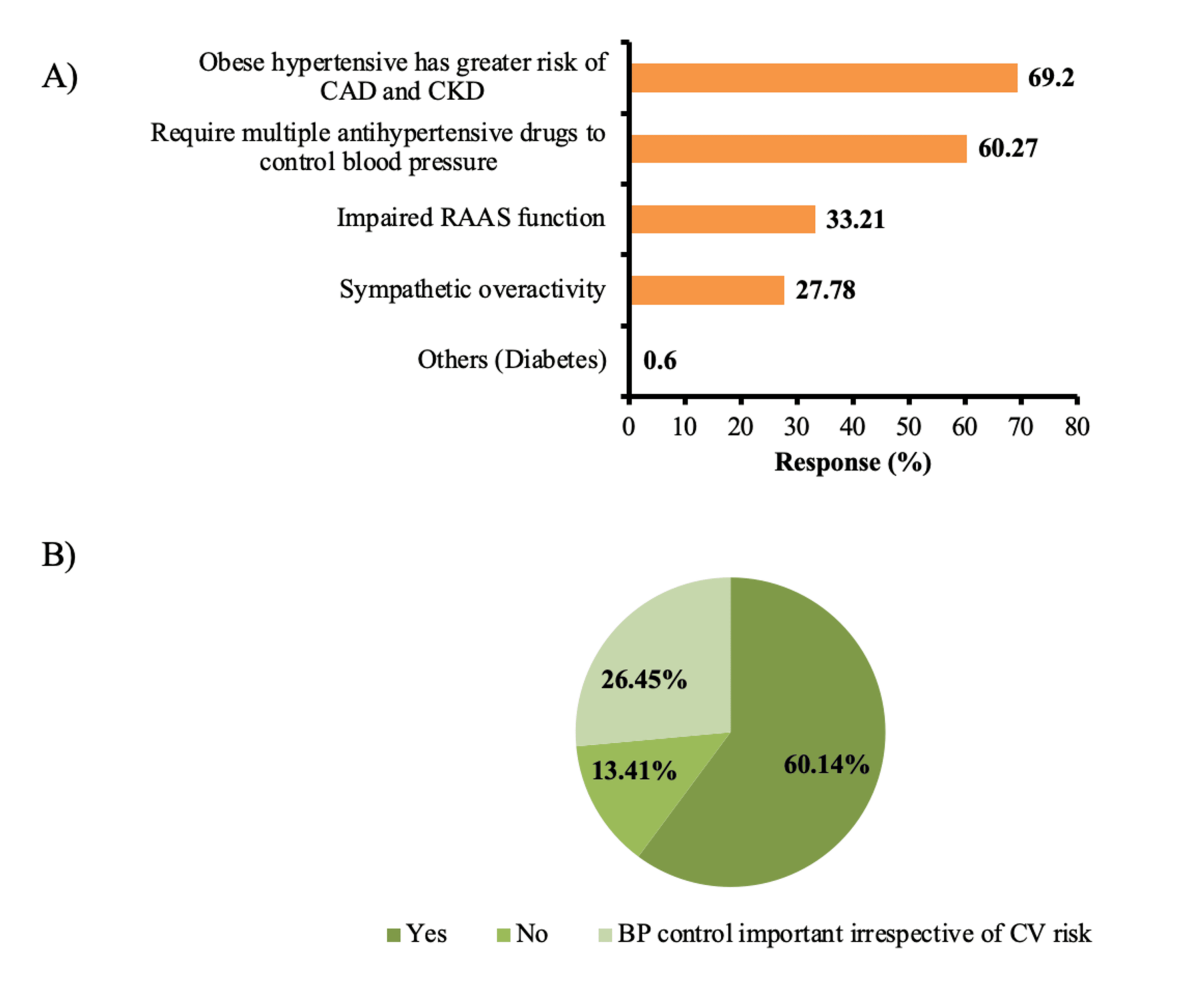
(A) Major challenges in the treatment of hypertension in obese. (B) Clinical approach for young adults with low cardiovascular risk. CAD: coronary artery disease; CKD: chronic kidney disease; RAAS: renin-angiotensin-aldosterone system; BP: blood pressure; CV: cardiovascular.

HCPs opined that obese patients with hypertension usually have a greater risk of coronary artery disease (CAD) and chronic kidney disease (CKD) (69%) and require multiple antihypertensive drugs (60.0%) (Figure 1A). However, 60% of HCPs recommended intensive blood pressure control to prevent future complications in young adults, even with low cardiovascular risk (Figure 1B).

Based on the discussion on the management among patients with hypertension and high sympathetic overactivity, telmisartan plus cardio-selective beta-blockers (n = 544, 66.0%) were preferred drugs (Table 2).

**Table 2:** Choice of drug combination for the management of sympathetic overactivity in patients with hypertension. Data are presented as n (%).

Drug combination	No. of doctors (N = 828)
Telmisartan + cardio-selective beta-blockers	544 (65.70)
Telmisartan + calcium channel blocker	178 (21.50)
Telmisartan + thiazide diuretics	78 (9.42)
Telmisartan + spironolactone	28 (3.38)

HCPs opined that a combination of telmisartan and metoprolol is considered in obese patients with hypertension to control sympathetic overactivity. Many HCPs noted that this combination is prescribed due to its potential to prevent structural and functional modifications of the myocardial tissue, as depicted in Table 3.

**Table 3:** Reasons for considering telmisartan and metoprolol combination in obese patients with hypertension. Data are presented as n (%).

Parameter	No. of doctors (N = 828)
Control sympathetic overactivity in obese patients	513 (61.96)
Preventive effect on structural and functional modification of myocardial	451 (54.47)
The combination does not alter the glucose and lipid level	210 (25.36)
Not recommended because of weight gain with a combination	73 (8.82)

## Discussion

Hypertension is common among Indian adults, particularly those between 20 and 40 years old [25]. There is a lack of guidance on effective management for this population. This expert viewpoint will provide insight into management strategies and the role of specific medications, i.e., telmisartan and metoprolol, for young patients with hypertension who have high sympathetic overactivity.

Hypertension and related cardiovascular disorders are linked to sympathetic overactivity [26], which is on the rise in India. Clinicians should be aware of symptoms, including restlessness, excessive sweating, and hand tremors, while treating patients with hypertension [19].

Experts suggest that the age range of 21-40 years is appropriate for sympathetic overactivity/stress, but hypertension is often diagnosed at 40-60 years of age. This may explain why a similar pattern of 41-60 years of age for sympathetic overactivity was observed in clinical practice [19]. However, it is essential to note that sympathetic overactivity can start as early as 20 years old, and early diagnosis can prevent further complications.

Obesity is an independent risk factor for hypertension and a well-known risk factor for CAD and CKD [27]. A joint statement of the European Association for the Study of Obesity and the European Society of Hypertension states that obese patients with hypertension require more antihypertensive medication than normal-weight patients with hypertension because they have a higher risk of cardiovascular disease due to their obesity [20]. Obesity is a known risk factor for cardiovascular disease, yet clinical guidelines do not fully address how it affects cardiovascular risk. Additionally, there is a lack of large-scale studies on the impact of severe obesity on morbidity and the effectiveness of antihypertensive medications in treating obese patients with hypertension.

A panel of experts was in favor of intensive blood pressure control for certain patient populations, such as young patients with good tolerability and those with a family history of hypertension. This is in line with the recommendations from the American Heart Association, which suggest that young patients with stage 1 hypertension and a low risk for cardiovascular disease, who are not achieving their target blood pressure, should consider intensive blood pressure control in combination with lifestyle modification [28].

The sympathetic nervous system plays a crucial role in maintaining cardiovascular health due to its key role in regulating blood pressure and blood flow to organs [29]. Therefore, if we target sympathetic overactivity, it will improve cardiovascular health in patients with hypertension. A panel of experts opined that telmisartan in combination with a cardio-selective beta-blocker is the most preferred antihypertensive treatment in patients with hypertension who have high sympathetic overactivity. The panelists suggested that using beta-blockers with telmisartan would be more effective for Indians with hypertension due to their higher heart rate than Caucasians.

Moreover, they emphasized that the combination of telmisartan and metoprolol is effective for treating sympathetic overactivity in obese patients with hypertension. This combination can also help prevent structural and functional changes in the myocardium, such as left ventricular remodeling [30]. When used appropriately, antihypertensive medications such as telmisartan and metoprolol can help reduce arterial and ventricular stiffness, improve ventricular-arterial coupling, reduce cardiac workload, and enhance left ventricular efficiency [31]. Guidelines suggest that beta-blockers alone or in combination with ARBs were more effective in reducing blood pressure in obese patients with hypertension than non-obese patients with hypertension [32,33]. HCPs have observed that the telmisartan/metoprolol single-pill combination was a well-tolerated combination therapy among young patients with hypertension [21]. However, their use is limited to young obese patients with hypertension without cardiac and renal complications [34]. Metoprolol is the most preferred cardio-selective beta-blocker due to its blocking effects on beta-1 receptors with minimal or no effects on beta-2 receptors. According to this expert viewpoint, HCPs believe that beta-blockers, such as metoprolol, are effective for treating hypertension in patients with sympathetic overactivity and sleep apnea. However, metoprolol may cause weight gain and elevated low-density lipoprotein levels, making it less preferable as a first-line treatment. To mitigate this potential side effect, a lower dose of metoprolol in combination with other anti-hypertensive drugs could be considered. This may minimize the chances of experiencing side effects related to the use of metoprolol.

This study has several limitations. Firstly, it depends on self-reported data from HCPs, which could lead to bias. Secondly, the cross-sectional nature of the study means it only captures data at one point in time, restricting the ability to determine causation or observe changes over time. Additionally, since the study was conducted solely in India, the findings may not be applicable to other regions or countries.

## Conclusions

According to HCPs, sympathetic overactivity is becoming increasingly prevalent in young adults with hypertension. To address this, the panel concluded that telmisartan, in combination with cardio-selective beta-blockers, is often prescribed for young patients with hypertension who have sympathetic overactivity or obesity. This combination is aimed at preventing future complications. Future studies could explore the efficacy of telmisartan and cardio-selective beta-blockers in diverse populations, with a focus on long-term cardiovascular outcomes. Additionally, public health initiatives should target early screening and intervention for hypertension in younger populations, particularly those at risk of sympathetic overactivity or obesity, to prevent long-term complications and improve overall health outcomes.

## Appendices

**Table 4:** Study questionnaire. CAD: coronary artery disease; CKD: chronic kidney disease; RAAS: renin-angiotensin-aldosterone system; CV: cardiovascular.

1. In your clinical practice, which of the following is the most common age group identified with sympathetic overactivity/stress? • 21-40 years • 41-60 years • >60 years
2. Which of the following clinical conditions is usually associated with increased sympathetic overactivity in hypertensive patients? • Stroke • Sleep apnea • Thyroid problem • Alcoholism • Anxiety
3. Based on your clinical practice, what are the major challenges in the treatment of hypertension in obese patients? (Tick multiple). • Obese hypertensive has a greater risk of CAD and CKD • Require multiple antihypertensive drugs to control blood pressure • Impaired RAAS function • Sympathetic overactivity • Any other_______________________
4. In your clinical practice, in young adults with low cardiovascular risk, do you consider them for intensive blood pressure control to prevent future complications? • Yes • No • Blood pressure control is important irrespective of CV risk
5. Which of the following drug combinations will you prefer for hypertension management in patients with sympathetic overactivity/stress? • Telmisartan + cardio-selective beta-blockers • Telmisartan + calcium channel blocker • Telmisartan + thiazide diuretics • Telmisartan + spironolactone • Any other _______________________
6. Based on your clinical practice, which of the following parameters do you consider for the telmisartan and metoprolol combination in obese hypertensive patients? • Control sympathetic overactivity in obese patients • Preventive effect on structural and functional modification of myocardial • Combination does not alter the glucose and lipid level • Not recommend because of weight gain with combination
